# The breeding systems and floral visitors of two widespread African dry forest species of ethnobotanical significance

**DOI:** 10.1371/journal.pone.0292929

**Published:** 2023-10-19

**Authors:** Christine Rose Coppinger, Dara A. Stanley

**Affiliations:** 1 School of Agriculture and Food Science, University College Dublin, Dublin, Ireland; 2 Earth Institute, University College Dublin, Dublin, Ireland; 3 West Lunga Conservation Project, North-Western Province, Zambia; Indian Institute of Science, INDIA

## Abstract

Forest products derived from woody trees, such as fruits, seeds, honey, wood and others, are important resources for supporting rural livelihoods. However, little is known about the breeding systems or floral visitors of trees that provide these resources, often due to the difficulty of accessing tree canopies. This study addresses key knowledge gaps from a data poor region, providing information on the breeding systems and contribution of biotic pollination to two trees abundant in south-central Africa, that provide forest product supports for rural livelihoods: *Julbernardia paniculata* (Benth.) Troupin and *Syzygium guineense* (Willd.) subsp. *barotsense* F. White (Fabaceae and Myrtaceae respectively). The breeding systems of these species were assessed by conducting controlled pollination experiments, and then measuring the effects on reproductive success to determine the degree of self-compatibility and pollen limitation. Floral visitors and their behaviour were observed to provide preliminary information on possible pollinator groups. *S*. *guineense* appeared to be self-compatible, while *J*. *paniculata* showed signs of both self-incompatibility and pollen limitation. Floral visitors of both species were dominated by bees, with native honeybees (*Apis mellifera*) providing the highest visitation rates. These insights provide the first steps for understanding the reproductive ecology of these key tree species and can help to inform sustained management and conservation aimed at protecting forests and supporting rural livelihoods, as well as broaden the understanding of the floral visitors, and contribution of biotic pollination to forest tree reproductive success.

## Introduction

Forests can be highly diverse, especially tropical forests which contain ~60% of all vascular plant diversity [1]. Forests provide a multitude of ecosystem goods and services, including facilitating large-scale processes including water, nutrient and carbon cycles on which terrestrial life depends, and also provide a range of forest products to society [1]. Although deforestation is a global issue, Africa had the highest net forest area loss between 2010 and 2020 with 3.94 million ha lost per year [1]. Approximately 20% of the globally significant sub-Saharan African dry forests (~1.73 billion ha) [2], have already been converted to agriculture [3]. This has implications for the ~505 million inhabitants of this area [4], many of whom are rural communities depending on these forests as a source of food, energy, materials and livelihoods [3, 4]. Despite their importance and imperilment, dry forests are under-researched and under-prioritised [3, 5, 6].

Biotic pollination is a key ecosystem service in forest habitats, as the majority (94% on average) of tropical plant communities, including forests, are pollinated by animals [7]. However, pollinators can often be a limiting resource for plant reproduction [8, 9], and this can be exacerbated by forest loss which can negatively impact pollinator populations [10–13] and pollination services to nearby croplands [14–16]. Fragmentation of forest habitats and increased distances between conspecific trees could reduce tree reproductive success [17–19], increase selfing rates and contribute to inbreeding depression [20, 21] and pollen limitation [18]. Pollen limitation is often a feature of plant communities and a number of factors can contribute to this trend including: the mating strategy of a plant, that is whether or not it depends on outcrossing [18]; the availability of pollinators, especially where habitat loss impacts pollinator populations [17, 22]; pollinator behaviour [18, 23]; land use and habitat fragmentation which affects the distances between individuals in the landscape thus affecting the mechanisms that ensure their pollination [18, 24, 25]. Understanding whether plants are affected by pollen limitation can therefore hint at the degree to which they are affected by these factors and can guide conservation interventions.

The contribution of biotic pollination to the reproductive success of many forest trees is still unknown. Most tropical tree pollination or breeding systems studies have been conducted in either the Americas [e.g. 26–30], or Asia Pacific regions [e.g. 31–35], and only a few studies have been done on African tree species [e.g. 36–40] with general canopy research in Africa lagging behind that of other continents [41] (see S1 Table in S1 Appendix). This may be in part due to the difficulty of accessing forest canopies [41, 42], which makes them among the least studied of the planet’s habitats [43, 44], although many important ecological processes, including pollination and reproduction, take place there [45, 46]. Understanding tree breeding systems and flower visitation is an important first step in assessing pollination requirements and likely pollinator groups, which has implications for the valuation of pollination services in forest systems and their conservation as well as the sustained provision of forest products to local communities.

Here, we investigate the breeding systems and floral visitors of two tree species in Zambian dry forest ecosystems; *Syzygium guineense* subs. *barotsense* (a relative of the clove tree *S*. *aromaticum*, family Myrtaceae) and *Julbernardia paniculata* (family Fabaceae, formerly subfamily Caesalpinioideae but now within Detarioideae). These species were chosen as they provide important forest products for local communities, and their breeding systems and floral visiting communities are currently unknown. Both these species occur within miombo-dominant Zambezian dry forest ecosystems where the study took place.

**Fig 1:**
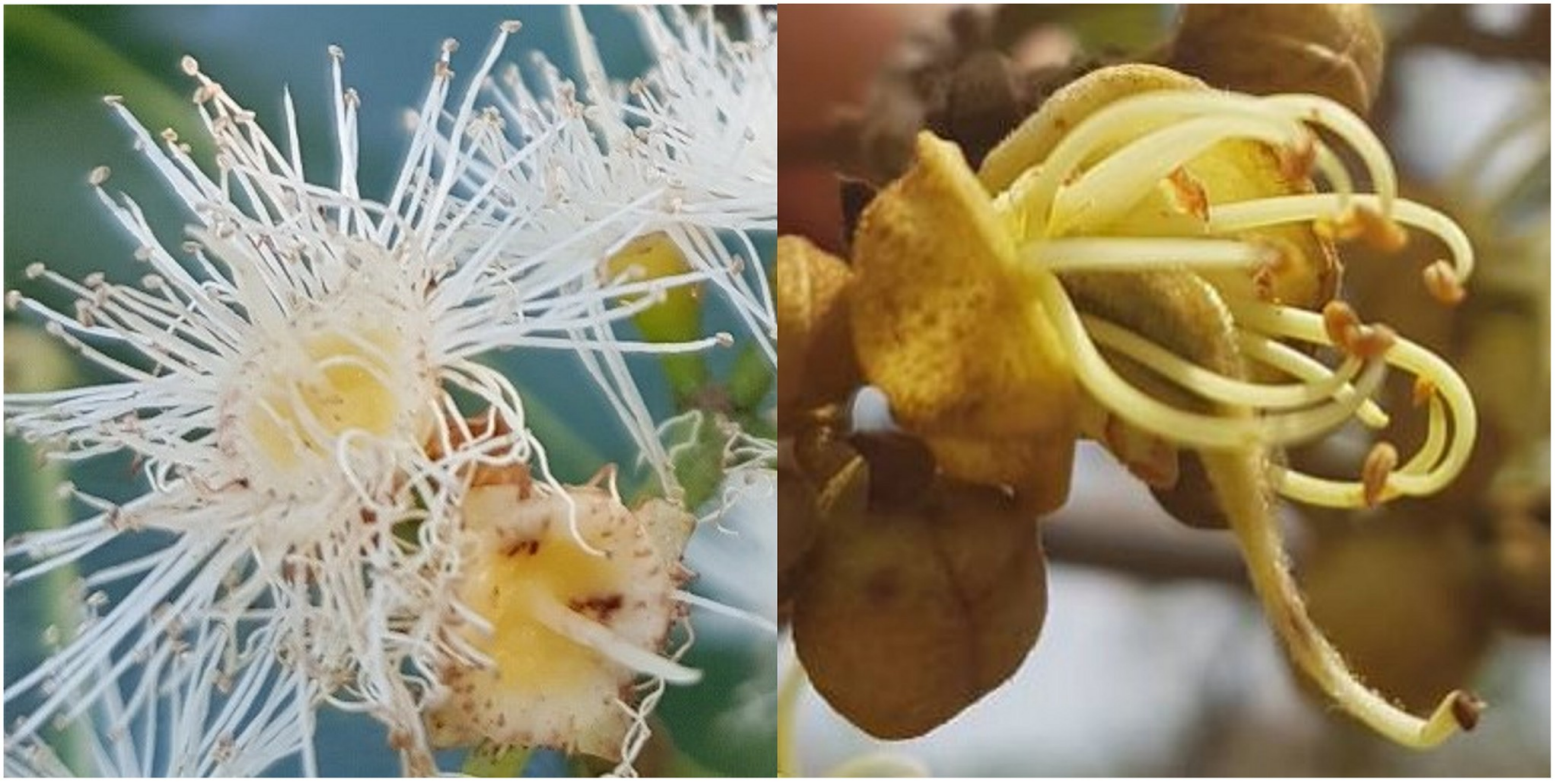
Flowers of the two Zambian miombo tree species selected for this study. Left: *Syzygium guineense* subs. *barotsense* (Myrtaceae) flower; Right: *Julbernardia paniculata* (Fabaceae) flower.

*Syzygium guineense* subs. *barotsense* (hereafter referred to as *S*. *guineense*) is a small (up to 13m tall) evergreen tree species that almost exclusively inhabits water body or water course margins and is largely restricted to the upper Zambezi River Basin [47] but with closely related subspecies occurring throughout the Zambezian region as well as in Tanzania, DR Congo, Angola, and South Africa [48]. This species can constitute the dominant riverine tree where it occurs. It generally flowers between August and September (late dry season), producing large displays of hermaphroditic, nectar-producing, sweet smelling white or cream flowers (1.5cm diameter) on panicles of roughly 18cm across [48] which are often showy and dominant in riverine and dambo habitats during this time (Fig 1, S1 Fig in S1 Appendix). *S*. *guineense* is presumed to be a significant nectar source for honeybees (*Apis mellifera*) supporting the production of honey [47]. The fruits are also sometimes utilised by people, and the bark and roots have medicinal properties [47]. Many studies have investigated reproductive aspects of other species within the more globally widespread and speciose *Syzygium* genus (~1200 species), finding predominantly at least partial self-compatibility but a range of reproductive strategies are employed within the genus including self-compatibility [32–34, 38], partial self-compatibility [31, 33, 35, 49], and self-incompatibility [50, 51]. A variety of floral visitors and pollinators have been documented for species within the *Syzygium* genus, including birds, bats, and a variety of insects such as bees, flies, moths, butterflies, wasps, ants, and others (see S2 Table in S1 Appendix).

*Julbernardia paniculata* is a large (up to 23m tall) semi-evergreen tree species andis the most common miombo tree species which is often dominant within plateau miombo woodlands and is distributed throughout the miombo region. The species is generally absent from drier miombo habitats (receiving <1000mm annually [52]) and is not found in Botswana or Zimbabwe [48]. *J*. *paniculata* flowers between March and June (late wet to early dry season in Zambia), producing hermaphroditic, nectar-producing, greenish-white, pleasant-smelling flowers (0.7cm diameter) in large, velvety terminal panicles [48] roughly 30cm across, and these displays often dominate the tree canopy (see Fig 1 and S2 Fig and S1 section in S1 Appendix). *J*. *paniculata* is a source of a wide range of wood and non-wood products used by rural communities including: building and tool materials and medicinal products [47, 48], traditional bark hives [4], charcoal [47, 53], and is a host species for several edible caterpillar species [47]. *J*. *paniculata* flowers have an open shape, similar to other Caesalpinioideae trees, making pollen and nectar easily available to a wide range of flower visitors [54], and it is also regarded as an important nectar source supporting honey production [47, 48, 53]. Most former Caesalpinioideae species have maintained self-incompatibility although this observed trend is based on only a few observations (Leiden and Larsen, 1996, see S1 Table in S1 Appendix for some examples). Legumes are in general thought to be pollinated by generalist bees [54], but floral visitors recorded within the former Caesalpinioideae include bees, butterflies, birds, and bats [55].

To contribute to an understanding of the reproductive ecology of these two tree species (*S*. *guineense* and *J*. *paniculata*), this study sought to determine: (a) their degree of self-compatibility; (b) whether they are pollen limited; (c) their main floral visitors; and (d) the behaviour of floral visitors (i.e., nectar versus pollen foraging). We addressed these questions by performing controlled pollination treatments on selected flowers of each species: manual self-pollination, manual cross- pollination, pollinator exclusion, and natural open-pollination. The reproductive success of treated flowers was then compared between these treatments, and floral visitors and their behaviour were observed. We hypothesise that these species will conform to general observed trends in terms of tree reproductive strategies: in other words, we predict that they will rely to some extent on outcrossing [56] and display some degree of self-incompatibility. We further hypothesise that these tree species may therefore be pollen limited, but that pollen limitation may in turn be influenced by reproductive strategy i.e. self-compatibility or incompatibility. We predict that bees will be the dominant floral visitors as has been found in many other pollination studies [e.g. 25] and that foraging behaviour will therefore include both pollen and nectar foraging.

## Materials and methods

### Study area

The study was conducted near Jivundu (13°06’04” S 24°41’37” E), adjacent to West Lunga National Park in North-Western Zambia. The habitat in this area is dominated by miombo woodlands, Zambezian dry evergreen forest, riverine forest, and floodplains [57]. Dry forests are widespread in Africa and characteristically experience prolonged dry periods, distinct seasonality, and high inter-annual climatic variability [5] and are likely to expand into previously humid forest regions as climate change continues [3]. Dry forests comprise roughly 40% of all tropical forests, harbour significant proportions of terrestrial carbon stocks [58, 59] and biodiversity [60], and are home to a considerable portion of the global population [3] many of whom are rural households vulnerable to a multitude of stressors [5]. Tropical dry forests globally face rapid deforestation [61] and numerous threats including agricultural expansion, fire [5, 62], and unsustainable forest product use [63]. Miombo forests are a type of dry forest which comprise of slow-growing, tall, closed-canopy, broad-leaved, deciduous forests, typically occur on nutrient-poor soils [52], and are dominated by the former Caesalpinioideae subfamily (now incorporated into Detarioideae). They extend from southern to central Africa and support millions of rural subsistence farming households, providing a safety net function in times of scarcity that are frequently brought on by crop failures as a result of intrinsic climatic variability, and provide myriad goods and services that are the foundation of informal rural economies [4].

### Breeding systems experiments

Trees of each species selected for the study were chosen within a small geographic area (~1.5 km^2^) to control for geographic differences in abiotic factors such as soil or climate between 2019 and 2020 and were selected to be as morphologically similar as possible i.e., generally similar in height and diameter at breast height (*S*. *guineense*: *n* = 14 trees, *J*. *paniculata*: *n* = 12 trees), and were all of a similar maturity stage. Both species have open, hermaphroditic flowers that are slightly different structurally (see Fig 1 and S1 section in S1 Appendix). Three replicate inflorescences (on average 10.57 ± 0.76 *SE* individual flowers per inflorescence for *S*. *guineense* and 2.13 ± 0.19 *SE* for *J*. *paniculata*) with predominantly unopened flowers, were selected for each of four treatments on each tree (3×4 inflorescences per tree, i.e. 3×4×14 = 168 treatment inflorescences for *S*. *guineense*, and 3×4×12 = 144 treatment inflorescences for *J*. *paniculata*), with each treatment being applied to a subset of flowers on separate inflorescences (i.e. only one treatment type was applied per treatment inflorescence). The treatments included: (a) a naturally open-pollinated treatment; (b) a pollinator exclusion treatment for which selected inflorescences were bagged with mesh bags (mesh size ∼1 mm^2^) to prevent pollinators accessing the flowers; (c) a self-pollinated treatment for which treated flowers were bagged before opening and then virgin flowers were pollinated manually with pollen from the same tree and then re-bagged (pollen was taken from a different flower on the tree); (d) a cross-pollinated treatment for which treated flowers were bagged before opening and then virgin flowers were manually treated with cross pollen from a different tree and then re-bagged (for consistency the same pollen donor tree was used for cross pollinations on all experimental trees). The number of flowers that could be hand-pollinated was limited by stigma receptivity at the time of treatment since hand-pollinations were only conducted once per treatment inflorescence, resulting in only a few flowers within each treatment inflorescence being treated. To avoid confounding effects of pollen dosage in the hand-pollinated treatments, treated stigmas were fully covered with treatment pollen. This additionally enabled assessments of pollen limitation to be made by comparing manually pollinated treatments (where the stigma received as much pollen as its surface allowed) to naturally open-pollinated treatments.

The number of flowers in the open-pollination treatment sometimes exceeded other treatments, as some flowers that were not yet stigma receptive were included in this treatment while the number of flowers that could be treated in the controlled pollination treatments was limited by the number of receptive flowers at the time of treatment. While this may have resulted in slight differences in age (a few days) of treatment flowers, we feel that the effect of this is negligible in the context of the four to five months allowed for fruit development. Pedicels of each individual treated flower within an inflorescence were marked with drab-coloured tags not attractive to pollinators (green, brown, black, grey) to identify the treatments and replicates within each treatment. Bags remained on the relevant treatment inflorescences until treatment flowers were no longer receptive to pollination: either flowers had been aborted, the stigmas had dropped off (e.g. the bottom *S*. *guineense* flower in Fig 1), or fruits had started forming. This ensured that these flowers received no additional biotic pollination (although small amounts of airborne pollen could potentially still reach the flowers through the mesh), but that fruits could develop normally without being affected by the bag. Flowers and fruit in the canopy were accessed using the doubled rope tree climbing technique [64, 65] by experienced tree climbers. Roughly the same number of days between treatment and fruit collection were allowed for development for all treated flowers (mean = 74.87 days ± 0.36 *SE* for *S*. *guineense* between October 2019 and January 2020; and mean = 117 days ± 0.70 *SE* for *J*. *paniculata* between May and September 2020). At the end of the experiment any resulting fruit from all treatments were harvested, fruit set and the number of seeds set counted, and fruits and seeds weighed to the nearest milligram. Seed number could not be recorded for *S*. *guineense* as it has single-seeded fruit, but all seeds were weighed. Trees and their habitats were not damaged during sampling and only a small fraction of fruits and seeds were sampled per study individual relative to their overall production.

### Floral observations

Floral observations were conducted for each species during flowering (in November 2018 for *S*. *guineense* and May 2019 for *J*. *paniculata*) to determine which insect taxa were the most common flower visitors, and whether visitors predominantly foraged for pollen or nectar (S1 and S2 Figs in S1 Appendix). Roughly the same amount of time was spent observing flowers of each species: 43 × 30-min observations were carried out for *S*. *guineense* (21.5 hrs total on 43 different trees), and 41 × 30-min observations for *J*. *paniculata* (20.5 hrs total on 41 different trees). Observations were stratified throughout the day with roughly equal numbers of observations conducted in each of three time-periods (0600 h– 1000 h, 1000 h– 1400 h, and 1400 h– 1800 h) to ensure comprehensive sampling across the day to minimise the effect of diurnal changes in floral visitors and their behaviour. The observer conducted focal sampling positioned ~1–1.5 m away from each patch of observed flowers to allow observation of small insects but still minimise impacts on flower visitor behaviour. Floral patches were selected that could be reached by the observer using the doubled-rope tree climbing technique and their size restricted to that which could be within the field of vision of the observer enabling all visitors to the patch to be seen, thus flower numbers within a patch varied depending on how densely flowers were clustered. Each individual 30-min observation was conducted in a different tree, and therefore on a different patch of flowers. The size of observation patch was kept as constant as possible between observations (on average 43.98 ± 0.37 *SE* flowers per patch for *S*. *guineense* and 20.35 ± 0.08 *SE* for *J*. *paniculata*). Data recorded included the number of visits made to observed flowers by each visitor, the total duration of the time they spent within the observed flower patch, and flower visitor behaviour: whether they collected pollen (P), nectar (N), or both (N+P) during their visit to the observed flower patch (see S1 and S2 Figs in S1 Appendix for images of these behaviours). Each flower visitor was identified to the lowest possible taxonomic level on sight and those that could not be identified were given morphospecies status until an individual of that group could be captured and identified in the laboratory. Captured insects were euthanised using ethyl acetate or frozen. Ethical approval was not required for insect research at the time of the study, however, the number of invertebrates lethally sampled for the purposes of more accurate identification in the laboratory was kept to a minimum, in line with ethical insect and animal sampling practices [66, 67], and floral visitors that could be identified in the field were observed in situ. No protected species were sampled. Additionally, care was taken not to damage habitats during fieldwork and all field materials were collected at the end of the experiments. The Zambia Ministry of Lands and Natural Resources provided permissions to carry out this research (reference number: MLNR/FD/101/8/7).

For analysis, taxa were grouped at the family level for bee groups other than the Apidae family which were grouped at species or genus level, and at the order level or just below for other insect groups, resulting in the following categories. Bee groups from the Apidae family included: *Apis mellifera*, *Meliponula*, *Hypotrigona* (all three in subfamily Apinae), *Braunsapis* (subfamily Xylocopinae), and bees in the families Halictidae and Megachilidae were observed. Other insect groups were wasps, ants, Coleoptera, Diptera, butterflies, and moths. For *J*. *paniculata Braunsapis*, Halictidae, and ants were not observed, but an additional two Apidae bee genera, *Amegilla* (subfamily Apinae) and *Xylocopa* (subfamily Xylocopinae), were included which were not observed on *S*. *guineense* flowers. Including bees with greater taxonomic resolution was done to account for the much higher visitation rates of bees when compared to other insect groups, and the greater diversity and higher visitation rates of bees from the Apidae family compared to other bee groups.

Climatic conditions (temperature, percent cloud cover, windiness, rainfall) and abiotic factors were kept as consistent as possible across observations. No observations were conducted under rainy or excessively windy conditions. The extent of exposure of observed flowers to the sky above and the position of the observed flowers within the canopy were kept as consistent as possible. As per the breeding system methods, flower observations were conducted within the canopy which was accessed using the doubled rope tree climbing technique. There were no managed beehives within this area and the nearest beehives (traditional or modern) were at least 5 km from any of the experimental trees at the time of the study, making it unlikely that managed honeybees would significantly influence the data as the areas surrounding the nearest hives are densely forested and provide relatively good forage. Additionally, all hives in the area receive minimal management (generally limited to bi-annual harvesting), are colonised naturally, and often have low occupation rates due to the frequent migration movements of the local wild native honeybees [68].

### Data processing and analysis

Data analysis and visualisation was conducted in R version 4.2.0 [69]. The ggplot2 package [70] was used for data visualisations, and the glmmTMB package [71] to run the generalized mixed-effects models. The DHARMa package [72] was used for model validation: the calculated residuals were used to plot the standard DHARMa qq-plot to detect overall deviations from the expected distribution with added tests for correct distribution (KS test), dispersion and outliers; as well as a plot of residuals versus predicted values with simulation outliers highlighted. Where model fit problems were detected further goodness-of-fit tests available in the DHARMa package were used. To quantify the magnitude of the differences between treatments for significant mixed models, the emmeans R package [73] was used to calculate Cohen’s *d* effect sizes and associated 95% confidence intervals. In cases where parametric model assumptions were not met, Kruskal-Wallis tests were conducted in R alongside pair-wise post-hoc analyses based on Bonferroni corrected Dunn’s tests using the rstatix [74] and tidyverse [75] packages.

#### Breeding systems

Mixed-effects models were used to test the effect of treatment on breeding success variables for each species individually, including fruiting success (binary), fruit weight, seed weight, and seed number (only for *J*. *paniculata* which has multi-seeded pods as *S*. *guineense* only produces one seed per fruit/ flower). As the experiment was replicated across multiple inflorescences within several trees for both species (12 *J*. *paniculata* trees, 14 *S*. *guineense* trees) and treatment used was always consistent within an inflorescence, inflorescence nested within tree was included as a random intercept for all models where replicates were fruits/seeds within inflorescence, since resources may be differentially allocated between inflorescences [76, 77]. For the binary fruiting success variable, treated inflorescence was the treatment replicate (either the inflorescence produced fruit or not: 1 or 0) and therefore inflorescence was not included in the random structure. Treated inflorescences that aborted all treated flowers were still counted as a fruiting failure (0) and therefore aborted flowers were considered in this variable. For fruit weight, seed number, and seed weight, individual flowers within inflorescences that were treated and that later produced fruit, or individual seeds within fruit in the case of seed weight, were taken as the treatment replicate (aborted flowers were not included for these variables or relevant analyses as measurements relied on actual fruits or seeds). There were smaller sample sizes for the fruiting success variable compared to the other variables in the *S*. *guineense* dataset as the abortion rate was relatively lower and since a single data point was always generated per treatment inflorescence for fruiting success (1 if inflorescences produced fruits and 0 if not) but all fruits and seeds produced by each inflorescence (sometimes multiple fruits were produced per inflorescence) counted as data points for the other variables. There were thus larger sample sizes for the fruiting success variable than the other variables for *J*. *paniculata* which had much higher abortion rates and some treatment inflorescences did not produce any fruit. Treatment was the fixed effect in all models.

#### Floral observations

Visitation rates were calculated for each taxon in each observation period as the total number of visits per flower per minute. Where a taxon wasn’t observed during an observation period (but was observed during another observation period), it was given a visitation rate of zero so each taxon had a value for each observation period. Floral visitor behaviours were assessed by calculating the visitation rates associated with each floral behaviour only for those taxa that were observed visiting flowers. The predominant floral behaviours were also assessed for each species by calculating both the frequency (the total number of visits when each behaviour was observed) and the duration (the total duration of all visits when each behaviour was observed) of each behaviour.

For each tree species, Kruskal Wallis tests were used to determine whether visitation rates were significantly different between (a) visitor taxonomic groups (zero inflation in the *J*. *paniculata* data was corrected for using a single zero inflation parameter applying to all observations), and (b) types of visitor behaviour. Skewed (non-normally distributed) visitation rate data that could not be remedied by data transformations prevented the use of parametric mixed models. Chi-squared tests were used to test differences in the frequencies of observed visitor behaviours, and Fisher’s exact tests were used to test the differences in frequencies of behaviours amongst the observed taxa as zeros for some of the category counts prevented the use of two-dimensional chi-squared tests.

## Results

### Syzygium guineense

#### Breeding systems

**Fig 2:**
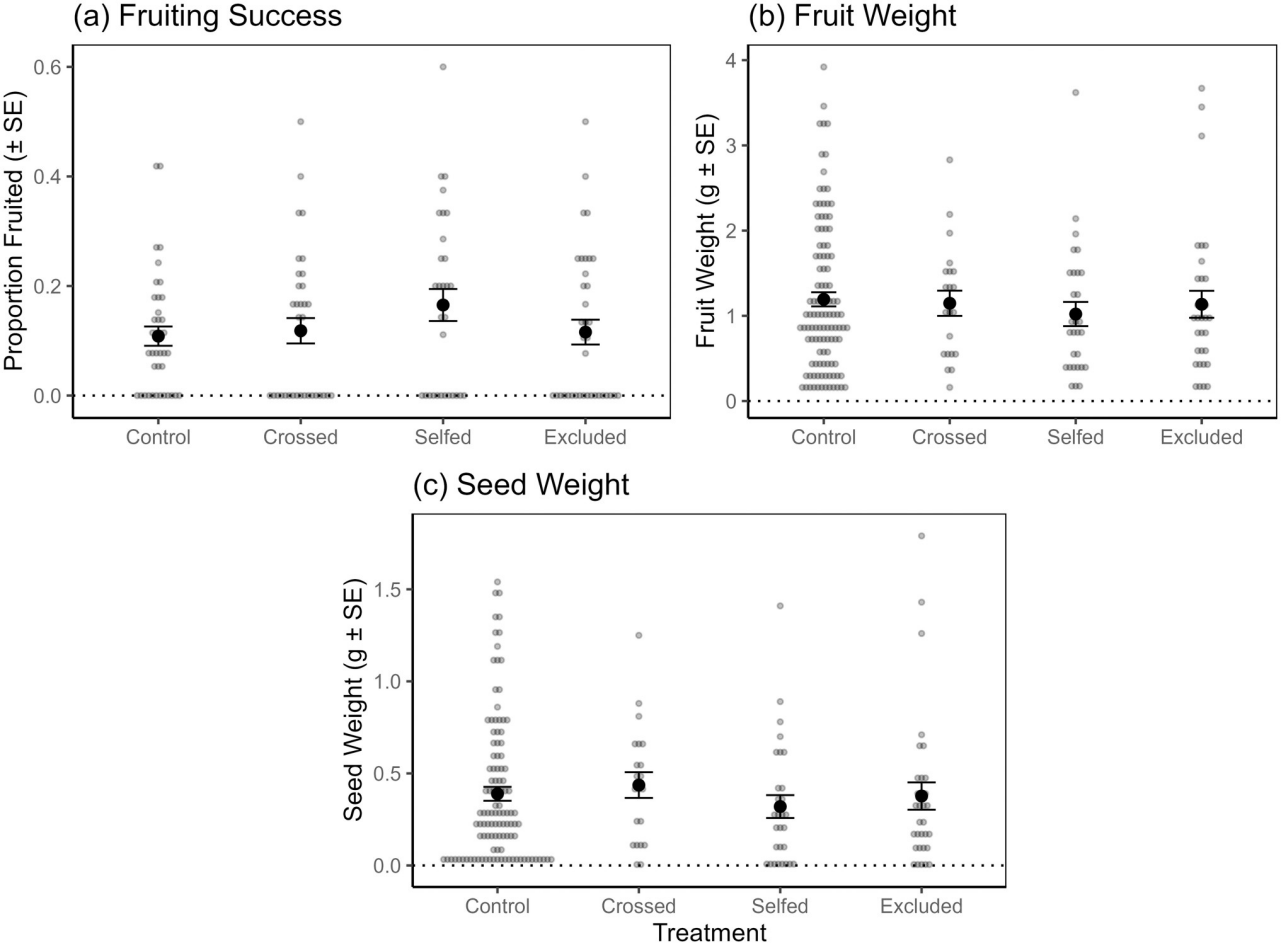
The effects of breeding system treatments on *S*. *guineense* breeding variables. (a) fruiting success rate (*n* = 142 flowers, including those that were subsequently aborted); (b) fruit weight (*n* = 193 fruit, aborted flowers excluded); and (c) seed weight (*n* = 173 seeds, aborted flowers excluded). Breeding system treatments were initially applied to 14 trees. Raw data points (treated inflorescences for (a) and treated flowers that produced fruits and seeds for (b), and (c)) are plotted in grey, while black dots and error bars show means and standard errors.

There was no difference in pollination treatments among breeding success variables (binary fruiting success GLMM: *χ*^2^ = 3.78, *p* = 0.286, n = 142, df = 3; fruit weight GLMM: *χ*^2^ = 0.34, *p* = 0.952, n = 193, df = 3; seed weight GLMM: *χ*^2^ = 0.88, *p* = 0.829, n = 193, df = 3) for *S*. *guineense*. This suggests that *S*. *guineense* is self-fertile and does not require cross pollination to produce fruits and seeds, although mean seed weight was slightly lower for the selfed treatment (Fig 2, S3 Table in S1 Appendix).

#### Floral visitation

**Fig 3:**
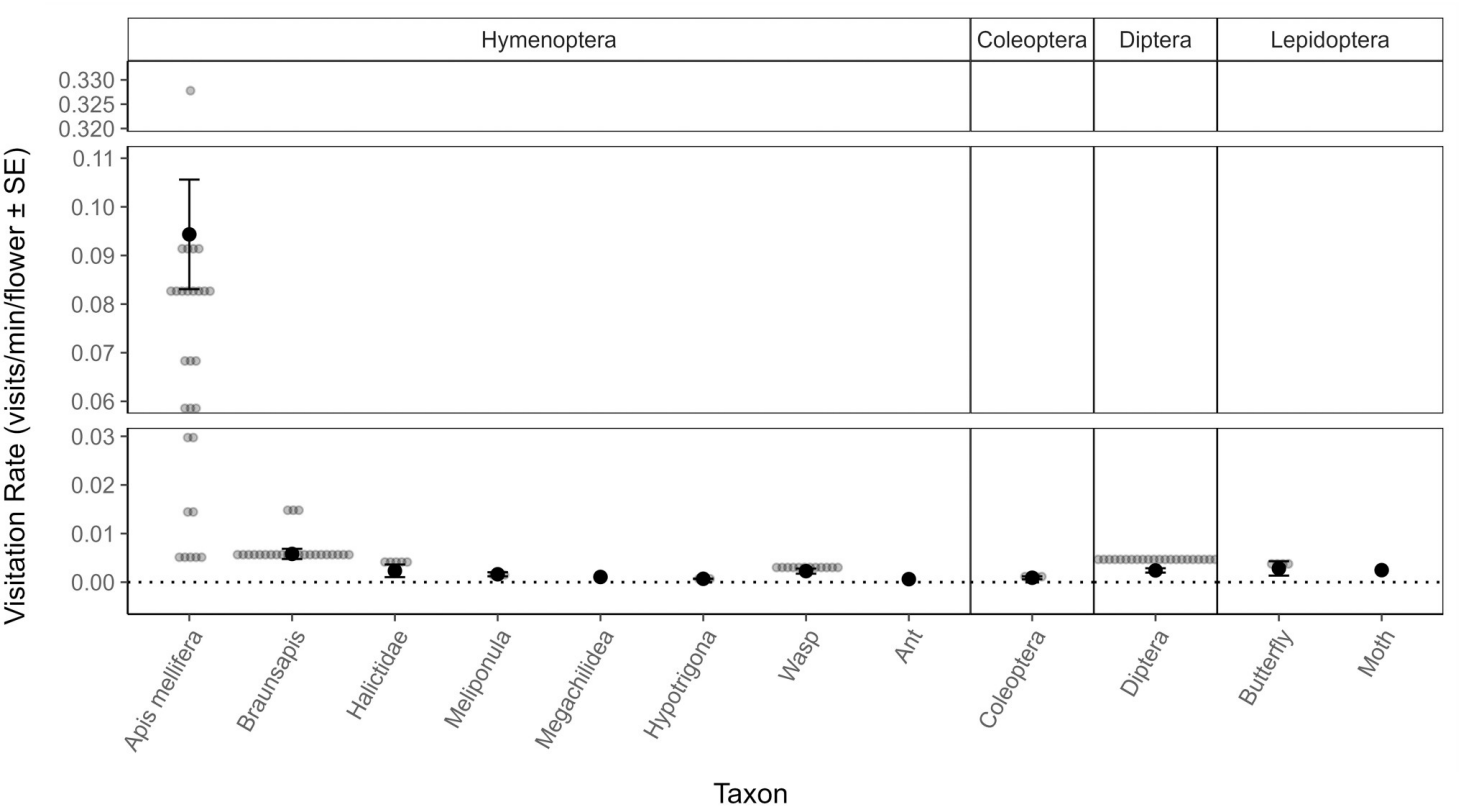
Mean visitation rates (visits /min /flower ± *SE*) to *S*. *guineense* flowers, for each of the floral visitor taxonomic groups recorded. Observations were conducted on 43 different trees. Bee groups (the first six taxa on the left) were included in more taxonomic detail while other insect groups were grouped at order level or just below, as bees were the main floral visitors. The plot is divided length-ways into insect orders which are shown at the top of the plot. Raw data points are plotted in grey. Two y-axis breaks were inserted (0.03–0.06 and 0.11–0.32) to enable easier plot interpretation, and raw visitation rate data points for *Apis mellifera* within these breaks are therefore not shown.

Insects from four orders (Hymenoptera, Diptera, Coleoptera, and Lepidoptera) were observed visiting *S*. *guineense* flowers, but bee groups, especially *A*. *mellifera*, were by far the most common visitors (Fig 3). Taxonomic groups visiting *S*. *guineense* flowers had significantly different visitation rates (Kruskal Wallis: *χ*^2^ = 259.36, *p* < 0.001, n = 516, df = 11): *A*. *mellifera* had significantly higher visitation rates than all other taxa; *Braunsapis* bees had higher visitation rates than all taxa except for *A*. *mellifera*, wasps, and Dipterans; and Dipterans had significantly higher visitation rates than all taxa other than *A*. *mellifera*, *Braunsapis* bees, and wasps. Although overall there was no significant association between visitation rate and floral visitor behaviour (Kruskal Wallis: *χ*^2^ = 0.56, *p* = 0.757, n = 144, df = 2), trends showed that nectar-foraging (N) visitation rates were generally higher than for the other behaviours observed (S3(a) Fig in S1 Appendix). Nectar foraging was the most common behaviour (Chi-squared test: *χ*^2^ = 3088.4, *p*<0.001, n = 1161, df = 3), and the greatest percentage of the total duration of floral visits was spent nectar foraging (S3(b) Fig in S1 Appendix: N duration = 92%), as were the greatest number of foraging visits (S3(c) Fig in S1 Appendix: N frequency = 95%). Nectar foraging by *A*. *mellifera* in particular was the most common (Fisher’s exact test: p = 0.001).

### Julbernardia paniculata

#### Breeding systems

**Fig 4:**
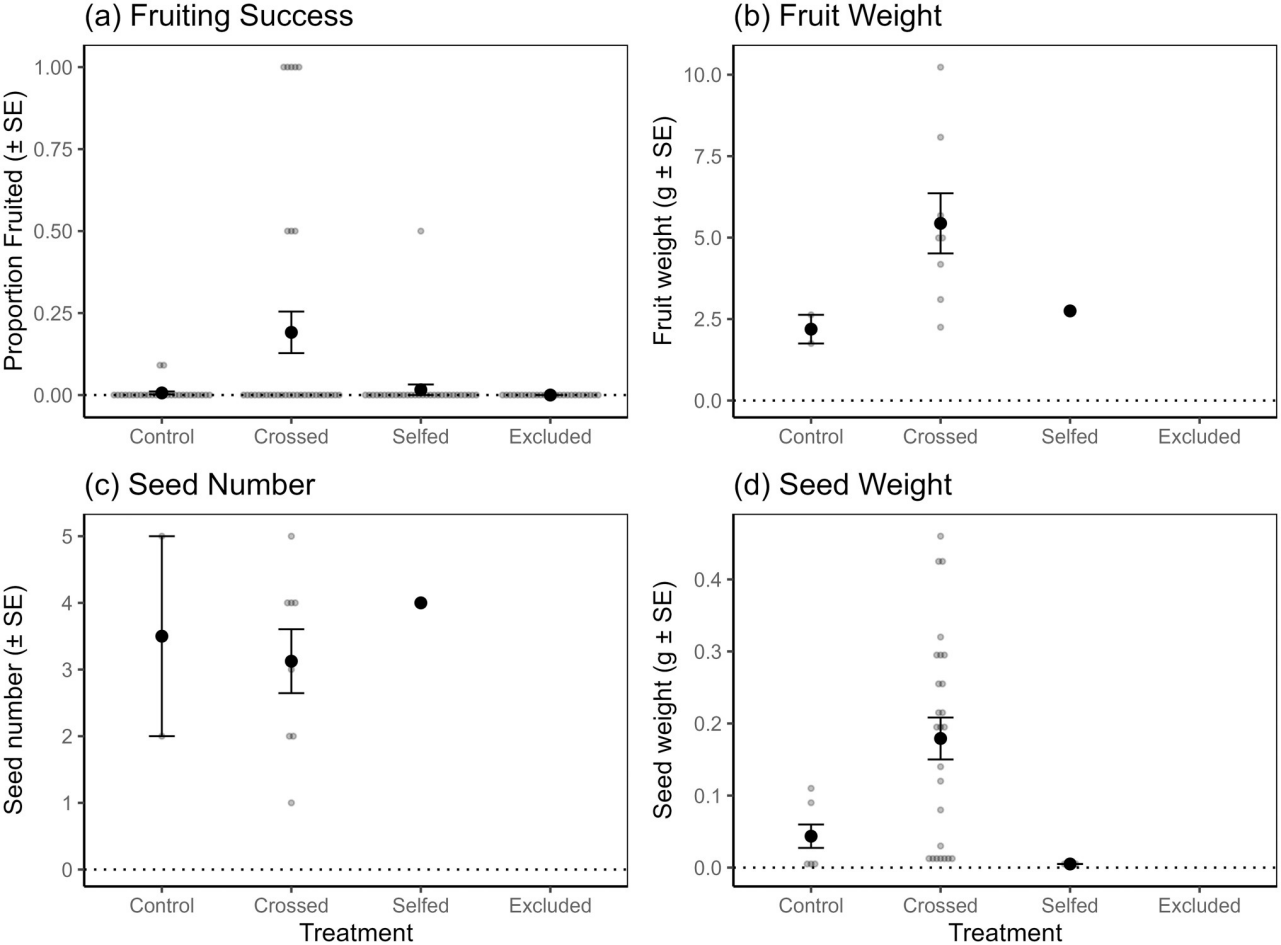
The effect of breeding system treatment on *J*. *paniculata* breeding variables. (a) fruiting success rate (*n* = 119 flowers, including those that were subsequently aborted), (b) fruit weight (*n* = 11 fruit, aborted flowers excluded), (c) seed number (*n* = 11 fruit, aborted flowers excluded), and on (d) seed weight (*n* = 36 seeds, aborted flowers excluded). Breeding systems treatments were initially applied to 12 trees. Means and standard errors are shown in black, while raw data points (treated inflorescences for (a), treated flowers that produced fruits and seeds for (b) and (c), individual seeds for (d)) are plotted in grey. Significant comparisons are shown with letters.

*Julbernardia paniculata* fruiting success was significantly affected by treatment (GLMM: *χ*^2^ = 12.99, *p* = 0.005, n = 119, df = 3), with the crossed treatment having the greatest proportion fruiting success compared with the other treatments (crossed: 0.19 ± 0.06 SE; selfed: 0.02 ± 0.02; control: 0.01 ± 0.01; pollinator excluded: 0.00 ± 0.00; Fig 4a). These results suggest that fruit set is reliant on cross-pollination. However, where fruits were produced, treatment had no effect on fruit weight (GLMM: *χ*^2^ = 3.93, *p* = 0.140, n = 11, df = 2), seed set (GLMM: *χ*^2^ = 0.24, *p* = 0.889, n = 11, df = 2), or seed weight (GLMM: *χ*^2^ = 5.34, *p* = 0.069, n = 36, df = 2), although trends indicated that the crossed treatment generally produced heavier fruits and seeds (Fig 4, S4 Table in S1 Appendix). Fruiting success being significantly greater for the crossed treatment than the open-pollinated control treatment indicates that the species might be affected by pollen limitation. Although there were no significant comparisons between treatments for the other variables measuring reproductive success, trends showed that the crossed treatment generally outperformed the open-pollinated treatment providing further evidence of possible pollen limitation. Fruiting success was generally low; many flowers were aborted in all treatments before fruits were formed, and the pollinator excluded treatment did not produce fruit.

#### Floral visitation

**Fig 5:**
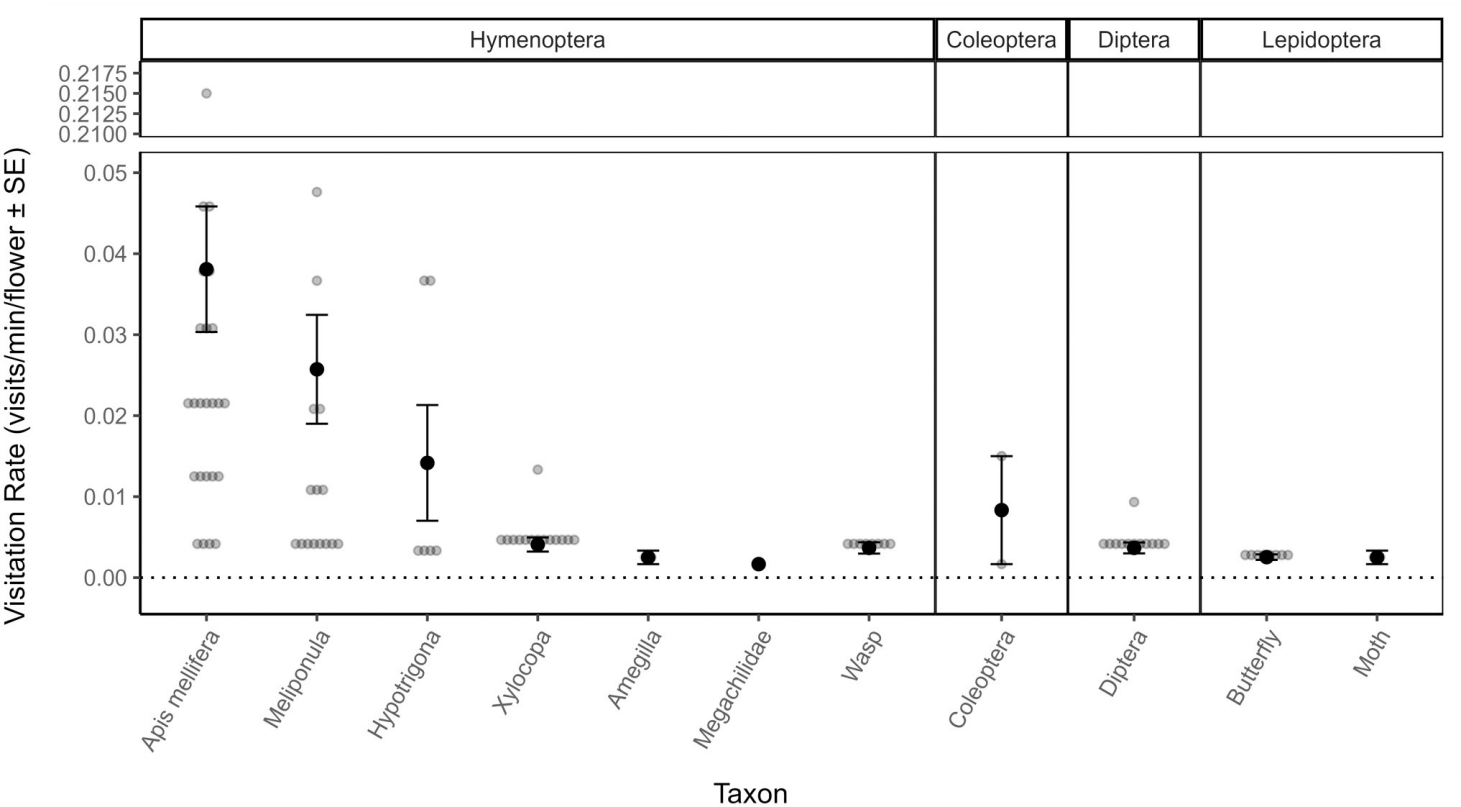
Mean visitation rates (visits/min/flower ± *SE*) to *J*. *paniculata* flowers for each of the floral visitor taxonomic groups seen visiting flowers. Observations were conducted on 41 different trees. Bee groups (first six taxa on the x-axis) were included in more detail while other insect groups were grouped at an order level or just below. The plot is divided length-ways into insect orders which are shown at the top of the plot. One y-axis break was inserted (0.05–0.21) to enable easier plot interpretation and raw visitation rate data points for *A*. *mellifera* within this break are therefore not shown.

Hymenoptera, Diptera, Coleoptera, and Lepidoptera were the four insect orders observed on *J*. *paniculata* flowers, with *A*. *mellifera*, followed by *Meliponula* bees, being the most frequent visitors with the highest visitation rates (Fig 5). Visitation rates were significantly different between the taxonomic groups (Kruskal Wallis: *χ*^2^ = 48.49, *p* < 0.001, n = 104, df = 10). *A*. *mellifera* had significantly higher visitation rates than wasps, Dipterans, butterflies, and Xylocopid bees. Overall, across all taxa, visitation rates differed significantly between the different types of visitor behaviour (Kruskal Wallis: *χ*^2^ = 22.1, *p* < 0.001, n = 147, df = 2): visitation rates associated with nectar combined with pollen foraging (N+P) were significantly higher than nectar foraging (N) visitation rates (S4(a) Fig in S1 Appendix), “All” taxa). The two most common floral visitors *A*. *mellifera* and *Meliponula* bees were most frequently observed foraging on pollen (P) and on both nectar and pollen combined (N+P) respectively (Fisher’s exact test: *p* = 0.001). The greatest duration of floral visits was spent nectar foraging (S4(b) Fig in S1 Appendix: N = 42%), while the greatest number of visits were spent on predominantly nectar but combined with pollen foraging (S4(c) Fig in S1 Appendix: N+P = 32%).

## Discussion

Consistent with many other studied legume species, *J*. *paniculata* appeared to be self-incompatible and reliant on cross-pollination as few fruit were developed by selfed flowers and none by pollinator excluded flowers, with the main flower visitors being social bee species within the Apidae family (primarily *A*. *mellifera* and *Meliponula* spp.). On the other hand, the *S*. *guineense* data suggested that this species is self-compatible which is relatively common within the *Syzygium* genus, although the viability of selfed seeds was not tested in this study. *S*. *guineense* floral visitors were similarly dominated by social Apidae bee species (primarily *A*. *mellifera* and *Braunsapis* spp.).

### Reproductive strategies and degree of self-compatibility

Although the *S*. *guineense* data suggests self-compatibility, spatial separation of the stigma and stamens in this species could indicate a preference for cross-pollination, as has been suggested for other self-compatible *Syzygium* species [e.g. 38] since outcrossing may still be desirable and may produce superior offspring [e.g. 36]. Although floral ontogeny was not comprehensively recorded during this study, there was an indication of some degree of dichogamy in *S*. *guineense* flowers, with the stigma often being erect (and therefore supposedly receptive) before full anthesis. Dichogamy has indeed been recorded for other Myrtaceae species [78]. This possible dichogamy for *S*. *guineense* cannot be confirmed, however, and the fact that pollinator-excluded flowers successfully produced fruits and seeds suggests that there may be some overlap of male and female flower functionality enabling autogamy, or apomixis may be occurring as has been found in other *Syzygium* species [34, 78], or some other mechanism enabling selfing. Further tests would be needed to confirm the exact mechanisms of selfing. There was no difference in fruit and seed production between manually selfed and pollinator excluded treatments, suggesting that efficient autogamous pollination in this species is possible and that autogamous pollination is just as effective in terms of fruit and seed production as geitenogamous pollination (manually selfed treatment), although further testing including emasculation experiments would be needed to rule out agamospermy as an avenue of self-pollination. The restriction of the species to riverine habitats, which are unevenly dispersed in the landscape, may be cause for the self-compatibility strategy, as individuals establishing in new areas may be too distant from the nearest population to ensure cross-pollination. Similar patterns have been found for species with similarly disjunct distributions [30], however further data on closely related species with similar floral biology and floral visitor profiles but differing distribution patterns would be needed to solidify this hypothesis for *S*. *guineense*.

The *J*. *paniculata* data appears, however, to corroborate the general trend of self-incompatibility in tropical trees [19, 26], with manually outcrossed flowers having significantly greater fruiting success and trends showing heavier fruits and seeds produced by outcrossed flowers. No seeds were produced by the pollinator excluded flowers, suggesting that this species is not adapted to be self-compatible, although further testing would be needed to rule out the possibility of self-compatibility. This result is unlikely due to an effect of the bag itself since excluded, selfed, and crossed flowers were all bagged for the full duration that flowers were receptive to pollination. Furthermore, the selfed treatment only produced a single fruit with vestigial seeds that may not have been fertile, suggesting that geitonogamous pollination may not result in breeding success. Two of the three species within the Detarioid subfamily within Fabaceae whose breeding systems have been studied elsewhere also demonstrate at least some degree of self-incompatibility: cross-pollination and open-pollination treatments outperformed self-pollinated flowers for *Tamarindus indica* and partial pre-zygotic self-incompatibility was demonstrated [37]; and *Guibourtia chodatiana* was found to be mainly outcrossing [28]. Further study is needed to confirm these findings for both species studied here, and this could include closer study of possible dichogamy; pollen tube growth assessment of self and cross pollen on stigmas; testing the viability of selfed seeds; or other methods to confirm the mechanism by which the respective compatibility strategy is achieved.

The abortion rate was high for *J*. *paniculata* in all treatments, which is relatively common among plants and tropical trees [76]. This could have been caused by a number of things [discussed by: 76, 79], for example lack of successful pollination, resource limitation [e.g. 36, 80], high levels of seed predation [e.g. 81], or variability in spatial and/ or temporal patterns of resource investment or selective abortion [e.g. 82]. Numerous herbivorous insects (Lepidopteran larvae and cocoons, and weevils) were observed around and on *J*. *paniculata* flowers even before fruit formation and therefore seed predation may have contributed to flower and fruit abortion. Outcrossing plants do, however, generally produce more flowers than they have the resources to develop into fruits and seeds whereas selfers have comparatively lower fruit:flower and seed:ovule ratios [76, 79, 83] as they may not be subject to the same constraints as outcrossers. Such constraints include the need to attract pollinators for which a large floral display is useful, or pollinator and subsequently pollen limitation [82]. Seed-set may therefore be a better indication of pollination efficiency, especially for self-incompatible trees [30]. Additionally, *S*. *guineense* may have been less resource-limited being a riverine species, which might have influenced the relatively lower abortion rates for this species [37, 76]. Another possible explanation is the differences in seed dispersal mechanisms between the species. *J*. *paniculata* relies on autochronous seed dispersal via explosive seed pod dehiscence possibly resulting in related individuals clustered in space and decreasing genetic diversity of crosses with nearby trees [e.g. 29]. On the other hand, *S*. *guineense* has fleshy, edible fruit, eaten by birds and mammals and are also often dropped into adjacent water courses and carried downstream, suggesting a possibly greater seed dispersal capability and greater genetic diversity among neighbouring trees.

### Pollen limitation

While fruiting success was reasonably high and there were no significant differences between treatments for *S*. *guineense*, fruiting success was comparatively lower overall for *J*. *paniculata*. The crossed treatment had the highest fruit set in this species suggesting self-incompatibility, also supported by a high abortion rate of flowers not pollinated with cross pollen, and fruit with vestigial seeds that were likely of low genetic quality from the selfed and pollinator excluded treatments [83]. A combination of selection promoting increased pollen dispersal as well as uncertainty in the paternity of zygotes could contribute to observed patterns of abortions in *J*. *paniculata* [76]. The crossed treatment resulted in greater mean fruiting success, fruit weight, and seed weight than other treatments including the open-pollinated control treatment (Fig 4, S4 Table in S1 Appendix) suggesting that this species may be affected by pollen limitation. These observations could also have been caused by high levels of self-pollination of open-pollinated flowers resulting from the behaviour of the dominant floral visitors, including *A*. *mellifera*, which tend to spend a large proportion of foraging time visiting flowers on the same plant rather than moving between plants. Given that the species appears self-incompatible, this high proportion of geitonogamous pollination could limit the availability of cross pollen and thus fruit set [10, 38, 84]. Such geitonogamous pollinations may be even more prevalent for plants with large floral displays such as many trees [e.g. 36]. On the other hand, *A*. *mellifera* have also been found to help in maintaining gene flow in fragmented and degraded habitats where their ability to move large distances while foraging can help to maintain connectivity [10]. Forest fragmentation, deforestation, selective exploitation, or otherwise increasing distances between conspecific plants, including apparently self-incompatible trees like *J*. *paniculata* that may be affected in this way due to destructive exploitation of this tree species for many wood products, could impact on pollination and possibly contribute to pollen limitation [23, 28], and result in inbreeding depression in severely affected areas [21].

### Floral visitors

Being the most frequent floral visitors, bees are likely to be important pollinators of both tree species, consistent with the general patterns for legumes [54], former Caesalpiniodeae [85], Myrtaceae [78], and tropical trees in general [7, e.g. 45]. The brush-flower morphology of *S*. *guineense* is associated with numerous and diverse floral visitors [sensu 23], however, for *S*. *guineense*, visits were dominated by *A*. *mellifera*. *Julbernardia paniculata* flowers have features considered consistent with melittophily: zygomorphic, sweetly scented, receptive during the day, and offering a nectar reward (although nocturnal pollination and flower receptivity was not assessed and was not within the scope of this study) [sensu 86]. High visitation rates may not necessarily be linked to a floral visitors’ effectiveness as a pollinator as higher visitation rates could translate to many visits on flowers of the same tree as opposed to between different individual trees, thereby increasing geitonogamous pollinations as previously discussed. Additionally, a floral visit does not necessarily imply that a pollination event has occurred due to other aspects that impact the effectiveness of floral visitors such as morphological fit, their effectiveness as pollen transporters, and whether pollen is effectively deposited on the stigma during a floral visit [87]. Pollinator effectiveness data is therefore needed to further explore which taxa are both visiting flowers and acting as pollinators, although this study provides a useful starting point for any such further research. When pollinator effectiveness data becomes available at the community level, this would allow the construction of interaction networks that would be more informative at contextualising the importance of these two tree species for pollinators and vice versa within the larger community.

### Floral visitor behaviour

Floral visitor behaviour was significantly dominated by nectar foraging by *A*. *mellifera* for *S*. *guineense*, consistent with the assumption that this tree species is an important nectar resource for *A*. *mellifera*, likely helping to support many honey-related rural livelihoods in areas where their distribution and beekeeping overlap. *S*. *guineense* flowering (August-September) coincides with a peak in general tree species flowering and thus *S*. *guineense* flowers must be particularly attractive since other nectar resources are presumably bountiful at that time. Indeed, other *Syzygium* species have been found to produce copious nectar [31, 78]. Contrastingly, *J*. *paniculata* appears to be important both as a pollen and a nectar resource for *A*. *mellifera* (and other social bees such as meliponines and hypotrigonids), suggesting that *J*. *paniculata* flowering could support bee colony growth and development as well as fuelling energetic demands. This presumably enables colonies to bolster their workforce and build reserves before the dry winter months (June-July) when there is a general dearth in flowering in the habitats studied [53]. Pollen foraging on the often dominant and mass flowering *J*. *paniculata* could thus explain the March-May *A*. *mellifera* swarming season in the region [53, 68], ultimately contributing to maintaining healthy populations and supporting gene flow and genetic diversity within *A*. *mellifera* [68] while also supporting honey production. The implications of nectar and/or pollen foraging for pollination service delivery would be determined by how effective the floral visitor is at contacting the sexual organs of the flower during these activities and whether pollen is actually transported or deposited [87]. This could be tested by examining floral visitor pollen loads and by conducting single visit deposition studies which was outside the scope of the current study. Although these aspects were not directly investigated, observations suggest that many of the bee visitors may effectively pollinate flowers of both species but may be more likely to do so during pollen foraging when they walk across the reproductive parts of the flower.

### Implications for forest products and services

Self-incompatible species tend to be negatively affected by habitat loss in terms of reproductive success [88] and therefore *J*. *paniculata* could be more impacted by habitat loss than *S*. *guineense*. Any impacts of habitat loss on *J*. *paniculata* are likely to increasing as deforestation continues and as the species continues to be targeted for wood-products (charcoal, firewood, traditional bark beehives) and destructively harvested for other forest products (e.g. extensive felling for edible caterpillar host species). This may result in inbreeding depression which in turn could reduce the fitness of progeny [21], reduce disease and pest resistance and further decrease fertility [sensu 28]. In this context, the conservation of floral visitors such as those recorded in this study which likely contain the pollinators that help to maintain connectivity and buffer against the effects of fragmentation, will be a particularly important consideration when developing forest conservation strategies. A study incorporating an investigation of key pollinators and the impact of fragmentation on pollination and tree breeding would be needed to confirm these hypotheses.

## Conclusions

The reproductive strategies of the two species studied can be summarised as follows: *S*. *guineense* being self-compatible and *J*. *paniculata* showing signs of self-incompatibility and pollen limitation. Although *S*. *guineense* appeared to be self-compatible, outcrossing may still be favoured and may still produce more successful offspring and future studies should investigate post-fertilization reproductive success. *Apis mellifera* and other bee taxa were dominant floral visitors to both species. While *A*. *mellifera* pollination could give rise to geitonogomous pollination due to their tendency to visit many flowers on the same plant rather than moving between plants (supported by our data which show high *A*. *mellifera* visitation rates), they are also known to maintain connectivity and increase genetic neighbourhood areas due to their ability to forage over long distances. Presuming that these and other long-distance floral visitors do effectively pollinate flowers of the tree species studied (preliminary observations of floral visitor behaviour suggests that this may be the case), this could be particularly important in the context of rapid forest loss and fragmentation for maintaining gene flow between forest patches. This is due to increasing distances between individuals in different forest patches that could reduce the chances of insect pollinators with smaller foraging ranges travelling between individuals and across breaks in forest habitat, thus reducing gene flow between distant forest patches. The reproductive strategies observed may also have implications for pollinator and thus forest conservation actions and the knock-on effects of these on the provision of forest products to rural communities who rely on them, as well as contribute to a better understanding of the role of biotic pollination to forest tree reproduction in the region.

## Supporting information

S1 Appendix Supplementary information. Additional information on breeding strategies and floral visitors of relatives of the studied species; images of floral visitors on *S*. *guineense* and *J*. *paniculata* flowers; summary statistics; and additional figures showing floral visitor trends.
(PDF)
