# Supplementary material for: The breeding systems and floral visitors of two widespread African dry forest species of ethnobotanical significance
Source: PLoS One. 2023 Oct 19;18(10):e0292929. doi: 10.1371/journal.pone.0292929 (PMC10586679; doi:10.1371/journal.pone.0292929)
Supplement: S1 Appendix — Additional information on breeding strategies and floral visitors of relatives of the studied species; images of floral visitors on S. guineense and J. paniculata flowers; summary statistics; and additional figures showing floral visitor trends. (PDF) [file pone.0292929.s001.pdf]

Appendix 1: Supplementary information

Manuscript: **The breeding systems and floral visitors of two widespread African dry forest species of ethnobotanical significance**

Christine R. Coppinger[a,b,c*], Dara A. Stanley[a,b]

[a] School of Agriculture and Food Science, University College Dublin, Dublin, Ireland

[b] Earth Institute, University College Dublin, Dublin, Ireland

[c] West Lunga Conservation Project, North-Western Province, Zambia

* Corresponding author: Christine Coppinger, christine@remoteafrica.com

Table S1: Reproductive strategies and floral visitors of studied species within the Fabaceae family. No information on pollination mechanisms or floral visitors was available for other species within the Africa-restricted *Julbernardia* genus, and so information from closely related species within the Fabaceae family within similar tropical habitats were included here.

**Species**	**Current Subfamily**	**Native range**	**Reproductive biology**	**Floral visitors (Diurnal/Nocturnal)**	**Reference**
Schizolobium parahyba<br>*Senna macranthera*<br>*Senna multijuga*	Caesalpinioideae	South America	Probably self-incompatible	50 bee species: medium-large Apidae species most frequent and important diurnal pollinators	[1]
Andira fraxinifolia<br>*Lonchocarpus cultratus*<br>*Pterocarpus violaceus*<br>*Swartzia oblata*<br>*Swartzia simplex*	Papilionoideae	South America			
Bauhinia forficata	Cercidoideae	South America	Predominantly self-incompatible, possible pollen limitation	Moths (nocturnal)	[2]
Parkia biglobosa	Caesalpinioideae	West Africa	Largely self-incompatible, possibly pollen limited	Bees (diurnal), bats (nocturnal)	[3]
Inga subnuda	Caesalpinioideae	Tropical Americas	Mixed pollination system	Bees, a wasp (diurnal), diurnal and nocturnal Lepidoptera, birds (hummingbirds) (diurnal), bats (nocturnal)	[4]
Pseudopiptadenia contorta	Caesalpinioideae	Tropical America	Self-incompatible	Native stingless bees, exotic honeybees (pollen collecting, no nectar) (diurnal)	[5]
Pseudopiptadenia leptostachya			Self-compatible		
Caesalpinia echinata	Caesalpinioideae	Tropical America	Late acting self-incompatibility	Bees: medium to large sized bees of genera *Centris* and *Xylocopa*, exotic *Apis mellifera* (diurnal)	[6]
Guibourtia chodatiana	Detarioideae	South America	Probably self-incompatible	Bees (*Trigona*, *Plebeia*, exotic *Apis mellifera*) (diurnal)	[7]

Tamarindus indica	Detarioideae	India/ Africa (widely introduced in last 400 years due to importance of fruits)	Partially self-incompatible	Social bees (*Apis mellifera*, *Polistes fastidious*, *Trigona* sp.), solitary bees (*Xylocopa olivacea*, *Megachile* sp.) (diurnal)	[8]
Colophospermum mopane	Detarioideae	Southern & central Africa	Possibly self-compatible but not confirmed	Presumably wind pollinated, but large numbers of mopane bees also seen collecting pollen (diurnal)	[9,10]

Table S2: Reproductive strategies and floral visitors of studied *Syzygium* species.

<table>
<tr><th>Species</th><th>Native range</th><th>Reproductive strategy</th><th>Floral visitors (Diurnal/Nocturnal)</th><th>Reference</th></tr>
<tr><td>S. syzygioides</td><td>From Northeastern India, through Thailand and Vietnam, Singapore to Indonesia</td><td>Self-incompatible</td><td>Short-tongued insects: Wasps, Dipterans (Syrphidae, Bombyliidae), Lepidoptera (Diurnal)</td><td>[11]</td></tr>
<tr><td>S. mamillatum</td><td>Mauritius</td><td>Possibly largely self-incompatible</td><td>Generalist birds: gray white-eye, red-whiskered bulbul, Mauritius bulbul (diurnal)</td><td>[12]</td></tr>
<tr><td>S. occidentale</td><td>Western Ghats, India</td><td>Partially self-compatible, considerable autogamy</td><td>Ants (Technomyrmex albipes), Xylocopa, Trigona, Apis cerana, birds (diurnal)</td><td>[13]</td></tr>
<tr><td>S. laetum</td><td rowspan="2">Western Ghats, India</td><td rowspan="2">Partially self-compatible</td><td>Birds (sunbird, white eye), Diptera (Syrphidae) (Diurnal)</td><td rowspan="2">[14]</td></tr>
<tr><td>S. mundagam</td><td>Birds (sunbird, green barbet), three bee species, three butterfly species
(Diurnal)</td></tr>
<tr><td>S. sayeri</td><td>Northern Australia</td><td>Partially self-compatible</td><td>Bats (nocturnal), Birds, Wasps, Flies, Thrips, Butterflies (diurnal)</td><td>[15]</td></tr>
<tr><td>S. cormiflorum</td><td>Australia</td><td>Partially self-compatible</td><td>Birds (diurnal), insects, bats, possibly large moths (nocturnal)</td><td>[16]</td></tr>
<tr><td>S. samarangense</td><td>Malay Peninsular & nearby islands</td><td rowspan="3">Self-compatible</td><td rowspan="3">Sunbirds, honeybees (Apis cerana), Ants, Butterflies (diurnal)</td><td rowspan="3">[17]</td></tr>
<tr><td>S. jambos</td><td>South-East Asia, Indonesia, the Phillippines, Malaysia</td></tr>
<tr><td>S. megacarpum</td><td>Assam to China and Indo-China</td></tr>
</table>

S. formosum	East Asia			
S. guineense macrocarpum	Benin	Self-compatible	Diurnal:<br>Diptera (26%), Hymenoptera (24%), Lepidoptera (19%), Orthoptera (11%), Coleoptera (10%), Hemiptera (7%)	[18]
S. tierneyanum	Australia	Self-compatible	Diurnal: Birds, Butterflies, Moths, Bees, Ants, Wasps, Diptera<br>Nocturnal: Bats, Moths<br>Feral *Apis mellifera* most common diurnal visitor	[19]
S. heyneanum	Western Ghats, India	Self-compatible	Insects: *Apis cerana* & *A. dorsata*, Halictidae, *Trigona* sp., (Diurnal)	[14]
S. travancoricum			Insects: Diptera (Syrphidae, Muscidae), Halictidae, *A. cerana & A. dorsata*, *Trigona*, Wasps, Butterflies (Diurnal)	

S1. Flower characteristics and floral visitors

Both *S. guineense* and *J. paniculata* have hermaphroditic flowers. *J. paniculata* has zygomorphic flowers with the stigma projecting at an angle beyond the eight to ten stamens and very small white petals mostly hidden within the brown velvety sepals which are retained during flowering; and *S. guineense* has open, petal-less, brush-shaped actinomorphic flowers with the stigma centrally orientated and many stamens with white filaments radially arranged in whorls around it (Fig S1). Both species' flowers have a pleasant, sweet-smelling scent, especially those of *S. guineense*.

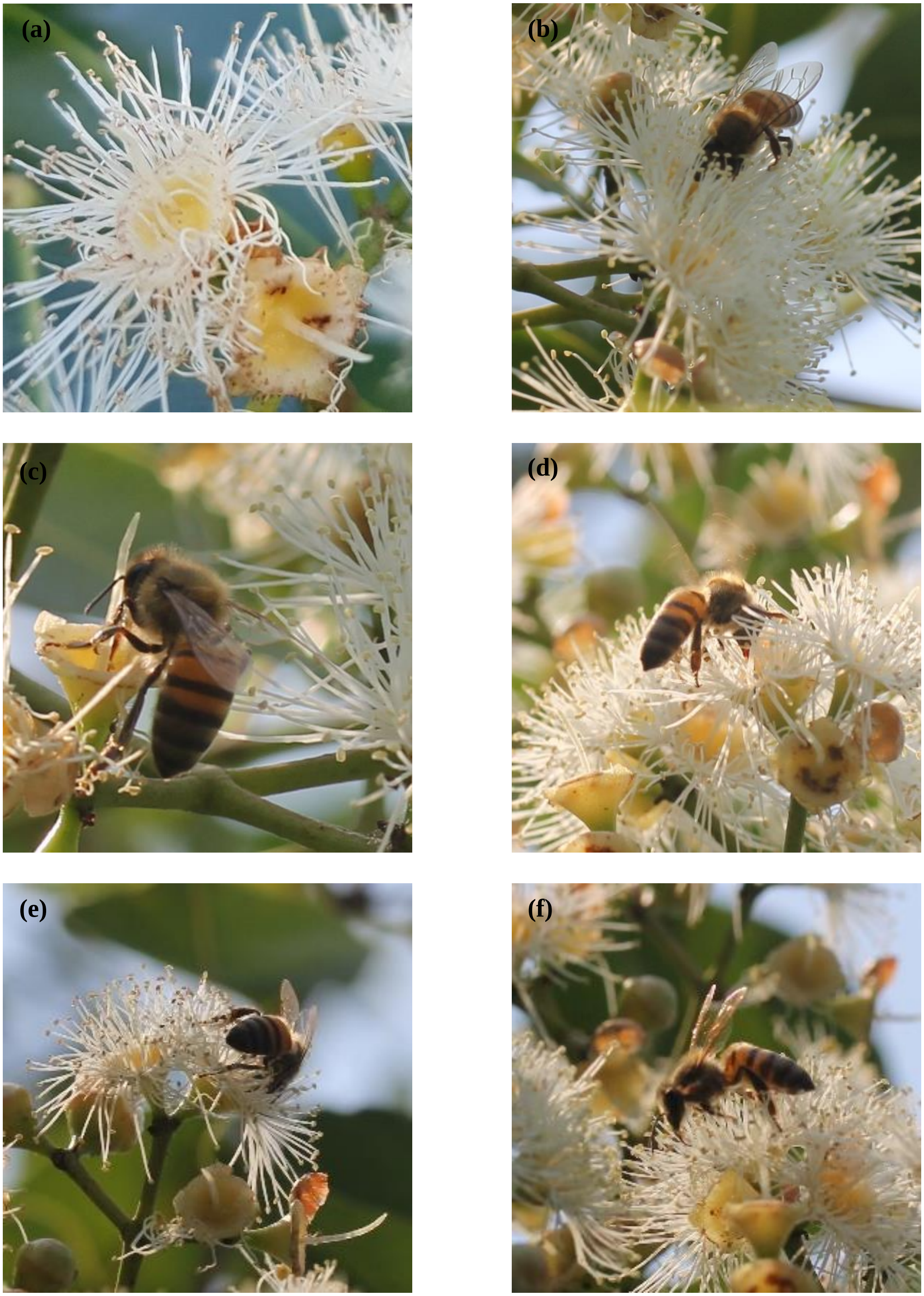
(a)
(b)
(c)
(d)
(e)
(f)

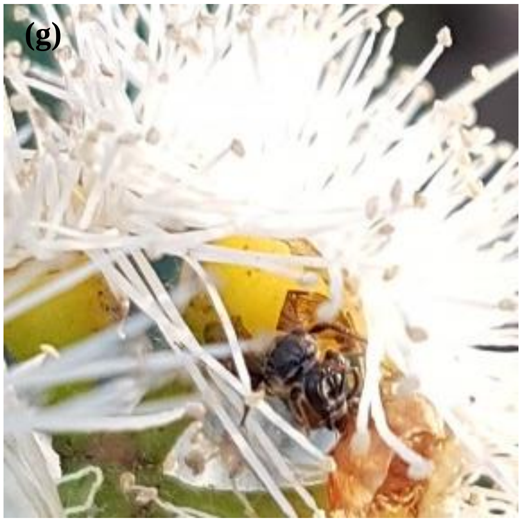

Fig S1: Images of *S. guineense* and some of the observed floral visitors. (a) *S. guineense* flower; (b) *A. mellifera* nectar foraging; (c) *A. mellifera* nectar foraging on a *S. guineense* flower after stamens have dropped off; (d) to (f) *A. mellifera* nectar foraging on *S. guineense*; (g) stingless bee amongst *S. guineense* flowers.

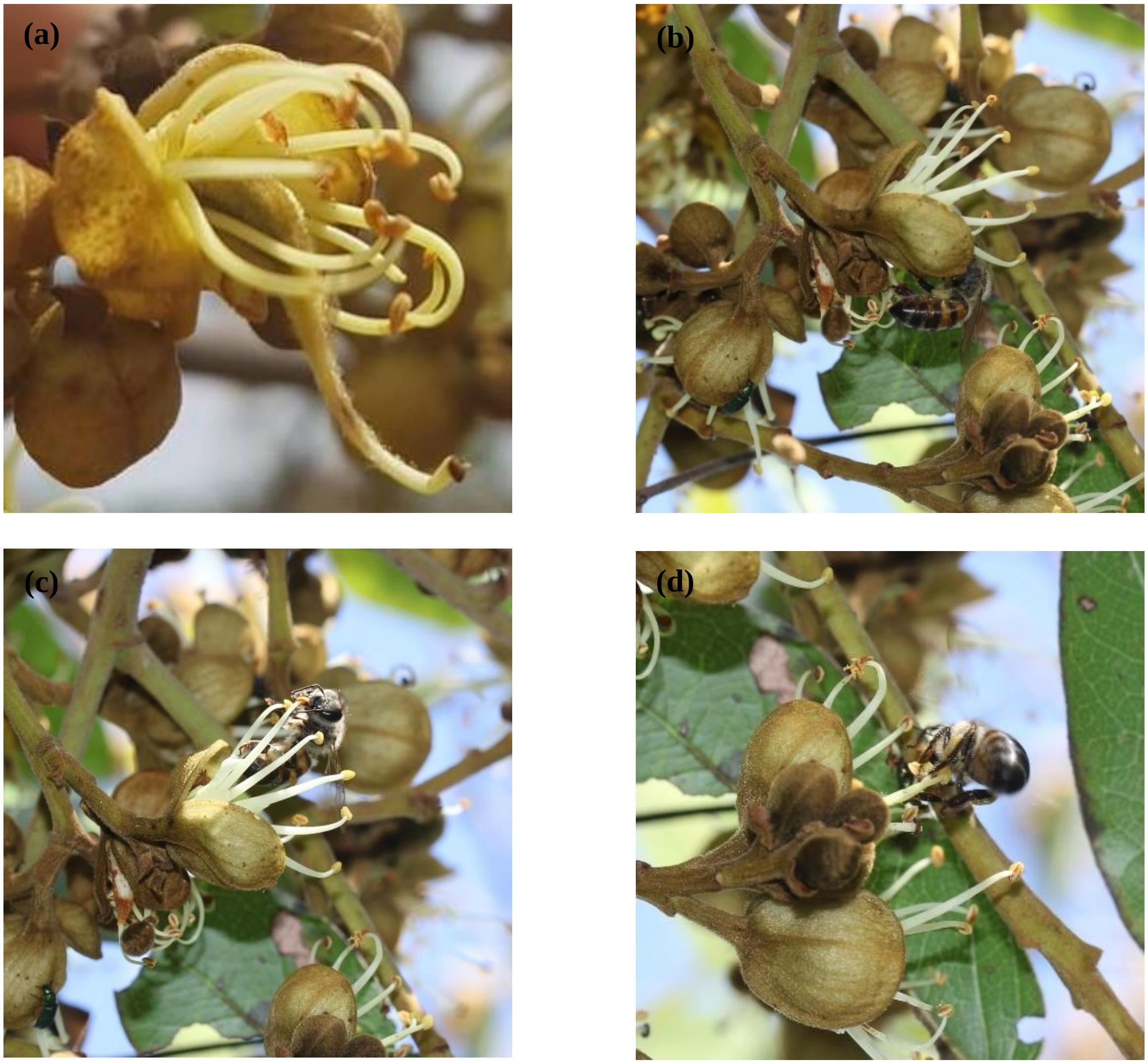
(a)
(b)
(c)
(d)

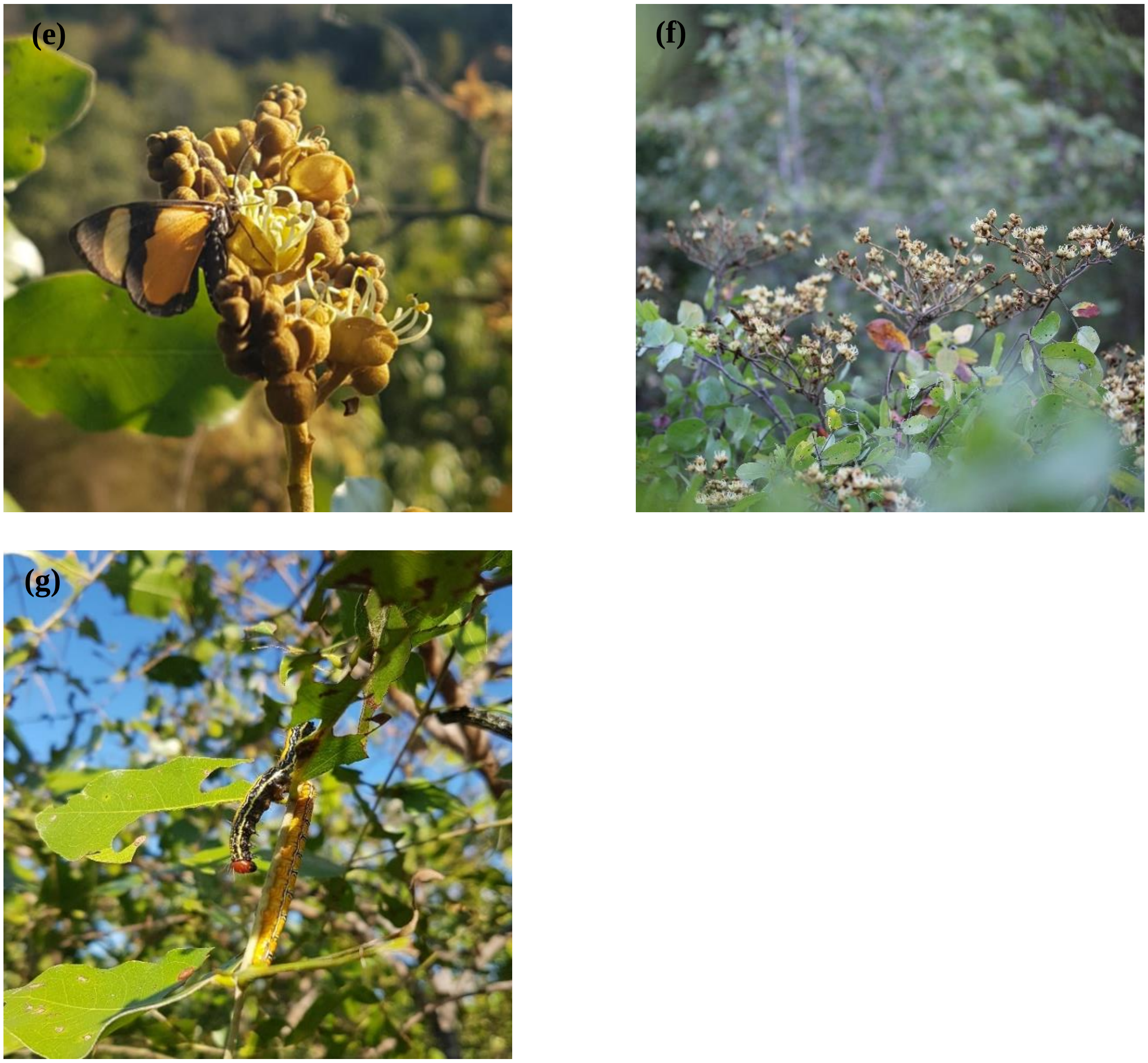

Fig S2: Images of *J. paniculata* and some of the floral visitors observed. (a) *J. paniculata* flower; (b) *Apis mellifera* nectar foraging on *J. paniculata* flower; (c) *A. mellifera* pollen foraging on *J. paniculata*; (d) *A. mellifera* pollen foraging on *J. paniculata*, making contact with stigma; (e) Lepidopteran nectar foraging on *J. paniculata*; (f) floral panicles of *J. paniculata* emerging from the top of the canopy; (g): edible satuurnid caterpillars feeding on *J. paniculata* leaves.

S2. Breeding systems, visitation rates and behaviour

As well as the models testing the relationship between visitation rate and all the taxonomic groups observed, two alternative taxonomic groupings or subsets were tested using Kruskal Wallis tests: (a) one with all bee groups pooled and another with (b) bees kept split but all non-bee groups removed from the analysis with results as follows.

Syzygium guineense

Summary statistics of the breeding systems results are shown in Table S3.

Table S3: Summary statistics for *S. guineense*: the total number of treated inflorescences per treatment, number of flowers treated per treatment, fruit and seed set per treatment and average weights of fruits and seeds per treatment. Inflorescence abbreviated as infl.

Treatment	Inflorescences treated (total *n*)	*n* of treated infl. that produced fruit	Flowers treated per infl. (mean± *SE*)	Fruit set (total *n*)	Proportion fruit set (mean ± *SE*)	Fruit weight (mean ± *SE*)	Seed weight (mean ± *SE*)
**Control**	39	27	23.60±1.88	113	0.11±0.02	1.19±0.08	0.40±0.04
**Crossed**	35	18	5.60±0.28	21	0.12±0.02	1.15±0.10	0.44±0.05
**Selfed**	31	19	5.76±0.26	29	0.17±0.03	1.02±0.11	0.32±0.05
**Excluded**	37	19	7.31±0.34	32	0.12±0.02	1.14±0.12	0.38±0.06

When bees were pooled into one taxonomic group there was still a significant difference in visitation rate among taxonomic groups (Kruskal Wallis: χ^2 = 175.68, p < 0.001, n=301, df=6): bees had significantly higher visitation rates than all other taxa, and Dipterans had higher visitation rates than ants, moths, Coleopterans, and butterflies. When only bee taxa were included in the analysis (as the visitation rates associated with non-bee taxa were comparatively very low), visitation rates were significantly different between bee groups: (Kruskal Wallis: χ^2 =

166.24, $p < 0.001$, n=258, df=5): honeybees (*Apis mellifera*) had higher visitation rates than all other bee groups, and *Braunsapis* bees had higher visitation rates than other bees except for honeybees.

Visitation rates associated with each of three behaviours (nectar foraging or N, pollen foraging or P, or both N+P) as well as the duration and frequency of each behaviour per taxon are visually depicted in **Fig S3**.

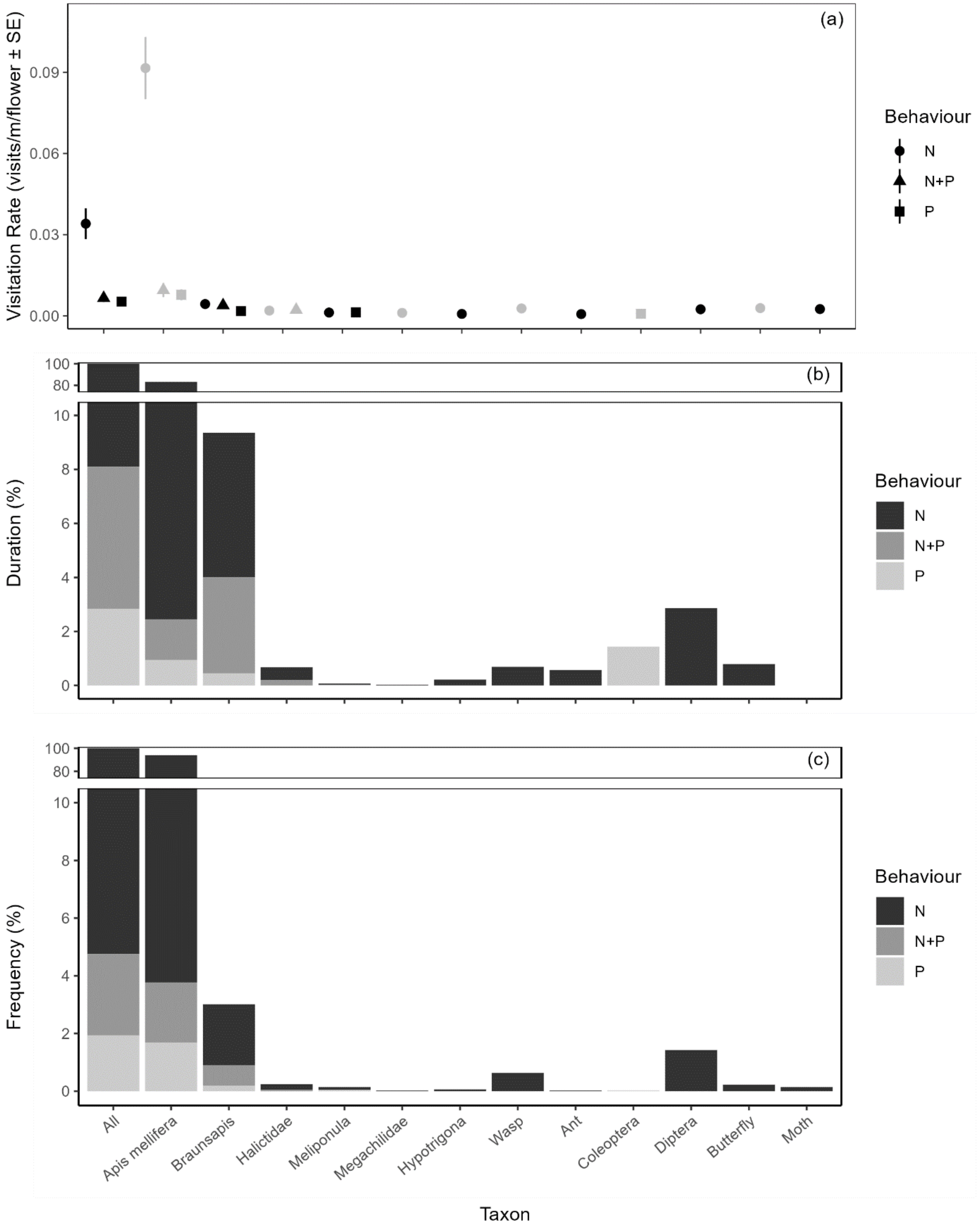

(a)
Visitation Rate (visits/m/flower ± SE)
0.09
0.06
0.03
0.00
Behaviour
N
N+P
P
(b)
Duration (%)
100
80
10
8
6
4
2
0
(c)
Frequency (%)
All
Apis mellifera
Braunsapis
Halictidae
Meliponula
Megachilidae
Hypotrigona
Wasp
Ant
Coleoptera
Diptera
Butterfly
Moth
Taxon

Fig S3: Floral visitor behaviour of visitors to *S. guineense* flowers. Floral visitor behaviour of visitors to *S. guineense* flowers (N = nectar foraging, N+P = nectar combined with pollen foraging, P = pollen foraging) on *S. guineense* flowers expressed as: (a) the visitation rate per behaviour type, per taxon and overall (points coloured alternately to enable different taxa to be easily distinguished); (b) the total observed duration of each behaviour per taxon and overall, expressed as a percentage of total visits overall (bars coloured by behaviour type, y-axis broken to enable small values to be visible); and (c) the number of visits where each behaviour was observed, calculated as a percentage of the total observed visits (y-axis broken to enable small values to be visible).

Julbernardia paniculata

Summary statistics of the breeding systems results are shown in Table S4 and effect sizes for the J. paniculata fruiting success model in Table S5.

Table S4: Summary statistics for *J. paniculata*: the number of treated inflorescences, mean number of flowers treated per treatment, the total numbers of fruit and seed that were set per treatment and mean individual weights of these fruits and seeds per treatment in grams.

**Treatment**	**Infl. treated (total *n*)**	***n* of treated infl. that produced fruit**	**Flowers treated per infl. (mean ± *SE*)**	**Fruit set (total *n*)**	**Proportion fruit set (mean ± *SE*)**	**Seed set (total *n*)**	**Seed set (mean ± *SE*)**	**Fruit weight (mean ± *SE*)**	**Seed weight (mean ± *SE*)**
**Control**	28	2	3.23±0.53	2	0.01±0.004	5	3.50±0.35	2.19±0.10	0.04±0.02
**Crossed**	34	8	1.19±0.09	8	0.19±0.06	19	3.13±0.23	5.44±0.43	0.18±0.03
**Selfed**	31	1	1.17±0.11	1	0.02±0.02	4	4.00±0.00	2.75	0.005±0.00
**Excluded**	26	0	2.86±0.45	0	0.00±0.00	NA	NA	NA	NA

Table S5: Proportion fruiting success per treatment for *J. paniculata* to support *J. paniculata* fruiting success model. The total number of inflorescences per treatment that were treated (n inflorescences); total number of treatment inflorescences that produced fruit (n inflorescences producing fruit); and the mean proportion of treatment flowers within treatment inflorescences producing fruit (Mean proportion fruiting success); and standard error (se) are reported.

Treatment	n inflorescences	n inflorescences producing fruit	Mean proportion fruiting success	se
**Control**	28	2	0.01	0.01
**Crossed**	34	8	0.19	0.06
**Selfed**	31	1	0.02	0.02
**Bagged**	26	0	0.00	0.00

When all bee taxonomic groups were pooled, visitation rates were still significantly different between taxonomic groups (Kruskal Wallis: $\chi^2 = 107.44$, $p < 0.001$, n=240, df=5) with bees having significantly higher visitation rates than other taxa. When all non-bee taxa, which had much lower visitation rates than most bee groups, were removed from the analysis, and visitation rate was compared between bee groups, there was a significant difference between the visitation rates of the bee groups (Kruskal Wallis: $\chi^2 = 87.04$, $p < 0.001$, n=240, df=5) with post-hoc tests showing that honey bees had significantly higher visitation rates than all other bee groups, and *Meliponula* had higher visitation rates than *Hypotrigona*, *Amegilla*, and Megachilidae.

Visitation rates associated with each of three behaviours (nectar foraging or N, pollen foraging or P, or both N+P) as well as the duration and frequency of each behaviour per taxon are visually depicted in **Fig S4**.

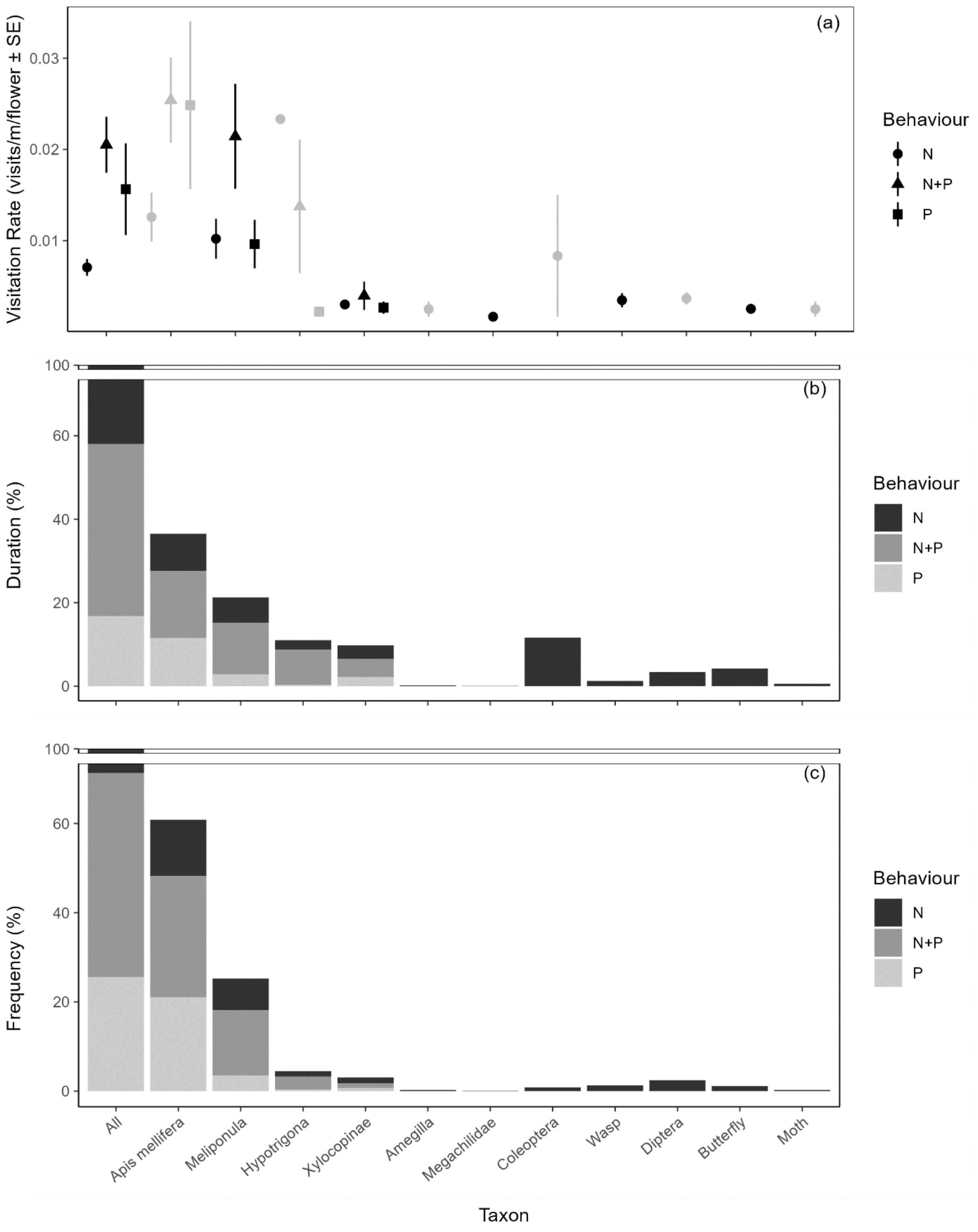
(a)
(b)
(c)
Visitation Rate (visits/m/flower ± SE)
0.03
0.02
0.01
Duration (%)
Frequency (%)
100
60
40
20
0
Behaviour
N
N+P
P
All
Apis mellifera
Meliponula
Hypotrigona
Xylocopinae
Amegilla
Megachilidae
Coleoptera
Wasp
Diptera
Butterfly
Moth
Taxon

Fig S4: Floral visitor behaviour of visitors to *J. paniculata* flowers. Floral visitor behaviour of visitors to *J. paniculata* flowers (N = nectar foraging, N+P = nectar combined with pollen foraging, P = pollen foraging) on *J. paniculata* flowers expressed as: (a) the visitation rate per behaviour type, per taxon and overall (points coloured alternately to enable different taxa to be easily distinguished); (b) the total observed duration of each behaviour per taxon and overall, expressed as a percentage of total visits overall (bars coloured by behaviour type, y-axis broken to enable small values to be visible); and (c) the number of visits where each behaviour was observed, calculated as a percentage of the total observed visits (y-axis broken to enable small values to be visible).

S3. References

1. Pinheiro M, Lourenço V, Brito G De. Pollination biology of melittophilous legume tree species in the Atlantic Forest in Southeast Brazil. Acta Bot Brasilica. 2018;32: 410–425. doi:10.1590/0102-33062018abb0078

2. Neto HFP. Floral biology and breeding system of *Bauhinia forficata* (Leguminosae: Caesalpinioideae), a moth-pollinated tree in southeastern Brazil. Rev Bras Bot. 2013;36: 55–64. doi:10.1007/s40415-013-0011-8

3. Lassen KM, Kjær DE, Ouédraogo M, Dupont YL, Nielson LR. Controlled pollinations reveal self-incompatibility and inbreeding depression in the nutritionally important parkland tree, *Parkia biglobosa*, in Burkina Faso. J Pollinat Ecol. 2018;24: 144–156. doi:https://doi.org/10.26786/1920-7603(2018)16

4. Avila Jr R, Pinheiro M, Sazima M. The generalist *Inga subnuda* subsp. luschnathiana

(Fabaceae): negative effect of floral visitors on reproductive success? Plant Biol. 2015;17: 728–733. doi:10.1111/plb.12291

5. Prata de Assis Pires J, Freitas L. Reproductive biology of two tree species of Leguminosae in a montane rain forest in southeastern Brazil. Flora. 2008;203: 491–498. doi:10.1016/j.flora.2007.10.002

6. Borges LA, Sobrinho MS, Lopes AV. Phenology, pollination, and breeding system of the threatened tree *Caesalpinia echinata* Lam. (Fabaceae), and a review of studies on the reproductive biology in the genus. Flora. 2009;204: 111–130. doi:10.1016/j.flora.2008.01.003

7. Ojeda-Camacho M, Kjær ED, Philipp M. Population genetics of *Guibourtia chodatiana* (Hassl.) J. Leonard, in a dry Chiquitano forest of Bolivia. For Ecol Manage. 2013;289: 525–534. doi:10.1016/j.foreco.2012.10.017

8. Diallo BO, Mckey D, Chevallier M, Joly HI, Hossaert-mckey M. Breeding system and pollination biology of the semi- domesticated fruit tree, *Tamarindus indica* L . (Leguminosae: Caesalpinioideae): implications for fruit production, selective breeding, and conservation of genetic resources. African J Biotechnol. 2008;7: 4068–4075.

9. Banks H, Rudall PJ. Pollen structure and function in caesalpinioid legumes. Am J Bot. 2016;103: 423–436. doi:10.3732/ajb.1500248

10. Jordaan A, Wessels DCJ, Krüger H. Structure of the style and wet non-papillate stigma of *Colophospermum mopane*, Caesalpinioideae: Detarieae. Bot J Linn Soc. 2002;139: 295–304. doi:10.1046/j.1095-8339.2002.00064.x

11. Lack AJ, Kevan PG, Kevan PG. On the reproductive biology of a canopy tree, *Syzygium syzygioides* (Myrtaceae), in a rain forest in Sulawesi, Indonesia. Biotropica. 1984;16: 31–36. doi:https://doi.org/10.2307/2387891

12. Kaiser CN, Hansen DM, Müller CB. Habitat structure affects reproductive success of the rare endemic tree *Syzygium mamillatum* (Myrtaceae) in restored and unrestored sites in Mauritius. Biotropica. 2008;40: 86–94. doi:10.1111/j.1744-7429.2007.00345.x

13. Kuriakose G, Allesh P, Shivanna KR. Ant pollination of *Syzygium occidentale*, an endemic tree species of tropical rain forests of the Western Ghats, India. Arthropod Plant Interact. 2018;12: 647–655. doi:10.1007/s11829-018-9613-1

14. Kuriakose G, Sinu PA, Shivanna KRA. Floral traits predict pollination syndrome in *Syzygium* species: a study on four endemic species of the Western Ghats , India. Aust J Bot. 2018;66: 575–582. doi:https://doi.org/10.1071/BT18042

15. Boulter SL, Kitching RL, Howlett BG, Goodall K. Any which way will do – the pollination biology of a northern Australian rainforest canopy tree (*Syzygium sayeri*; Myrtaceae). Bot J Linn Soc. 2005;149: 69–84.

16. Crome FHJ, Irvine AK. “Two bob each way”: the pollination and breeding system of the Australian rain forest tree *Syzygium cormiflorum* (Myrtaceae). Biotropica. 1986;18: 115–125.

17. Chantaranothai P, Parnell JAN. The breeding biology of some Thai *Syzygium* species. Trop Ecol. 1994;35: 199–208.

18. Badou RB, Yedomonhan H, Ewedje E-EBK, Dassou GH, Adomou A, Tossou M, et al.

Floral morphology and pollination system of *Syzygium guineense* (Willd.) DC. subsp. macrocarpum (Engl.) F. White (Myrtaceae), a subspecies with high nectar production. South African J Bot. 2020;131: 462–467. doi:10.1016/j.sajb.2020.04.013

19. Hopper SD. Pollination of the rain-forest tree *Syzygium tierneyanum* (Myrtaceae) at Kuranda, northern Queensland. Aust J Bot. 1980;28: 223–37.
